# The Role of Environmental Exposures in Pediatric Asthma Pathogenesis: A Contemporary Narrative Review

**DOI:** 10.3390/children12101327

**Published:** 2025-10-02

**Authors:** Luca Pecoraro, Anna Gloria Lanzilotti, Marta De Musso, Elisabetta Di Muri, Fernanda Tramacere, Emiliano Altavilla, Flavia Indrio

**Affiliations:** 1 Pediatric Unit, Ospedale Vito Fazzi, ASL Lecce, 73100 Lecce, Italy; 2 Pediatric Department, University of Bari Aldo Moro, 70121 Bari, Italy; 3 Department of Experimental Medicine Pediatric Section, University of Salento Hospital “Vito Fazzi”, 73100 Lecce, Italy

**Keywords:** pediatric asthma, environmental risk factor, tobacco smoke, air pollution, allergens, viral infection, gut microbiome, obesity, diet, sedentary lifestyle

## Abstract

Over several decades, childhood asthma has emerged as a significant global public health concern, with the highest prevalence reported in industrialized countries. The rapid rise in asthma incidence and loss of control when the diagnosis is established can be related to environmental and lifestyle changes, especially during early infancy. Current evidence indicates a potential link to an imbalance in immune system responses, influenced by tobacco smoke, traffic-related air pollution, outdoor and indoor allergens, gut microbiome, viral infection, obesity, sedentary lifestyle and dietary patterns. This narrative review aims to explore the landscape of contemporary environmental risk factors for childhood asthma, with a focus on their interplay and the relative importance.

## 1. Introduction

**Figure 1:**
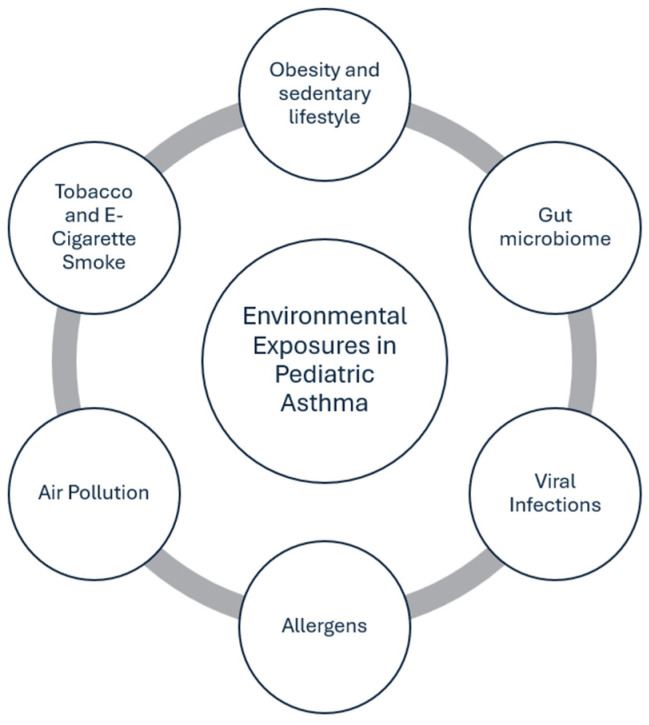
Environmental risk factors for pediatric asthma.

Over the past two decades, substantial epidemiological and clinical evidence has linked multiple environmental risk factors to pediatric asthma. These include outdoor air pollution (e.g., traffic emissions, particulate matter, nitrogen dioxide, ozone), indoor pollutants (e.g., tobacco smoke, mold, volatile organic compounds), allergen exposure (e.g., dust mites, pet dander), and early-life infections [1,2] (Figure 1).

The hygiene hypothesis remains a central paradigm in asthma research [3], suggesting that reduced microbial and antigenic exposure during early immune development impairs regulatory T cell (Treg) function—particularly IL-10 (Interleukin 10) and TGF-β (Transforming Growth Factor-beta)—thereby favoring a Th2-skewed immune response and increasing susceptibility to asthma and atopic diseases [3]. This may account for lower asthma rates in environments with high microbial load, such as rural areas or settings with helminth exposure and endotoxins [4]. Emerging challenges amplify environmental risk and disease burden, including climate change, urbanization, and socioeconomic disparities. Asthma’s natural history is heterogeneous and complex to predict [2]. Childhood-onset asthma has a greater chance of remission, whereas mortality is rare in the absence of comorbidities [5,6]. Longitudinal studies indicate that approximately 75% of school-aged children who wheeze in early life become asymptomatic by adulthood, although most chronic asthma cases begin within the first six years [2,7,8]. Specifically, infants with early respiratory infections often exhibit reduced lung function even before symptom onset [9,10,11]. The frequency and severity of early symptoms may also influence long-term outcomes. A 42-year longitudinal study of 317 children with wheezing found that 86% of those with infrequent symptoms at age seven had minimal or no asthma in adulthood. In contrast, 71% of those with frequent wheezing remained symptomatic [12,13]. The Childhood Asthma Management Program (CAMP), which followed 909 children aged 5–12, identified atopy, demonstrated reduced lung function, and airway hyperresponsiveness as predictors of persistent asthma [14]. Sensitization and exposure to indoor allergens tripled the risk of long-term asthma [14]. Globalization has facilitated the widespread convergence of asthma risk factors, including escalating urbanization, deteriorating air quality, dietary shifts toward high-calorie nutrient-poor foods, and sedentary lifestyles [15]. These factors have amplified the reach of asthma, extending its impact to regions historically less affected [15]. Concurrently, asthma continues to compete with other chronic and infectious diseases for healthcare resources, limiting its recognition as a priority in public health priorities [15]. According to the GINA 2024 update, environmental risk factors are also related to asthma control. Identifying and reducing environmental risk factors should precede any escalation in treatment [1,2]. While some risk factors are genetic, environmental ones are modifiable and thus critical intervention targets. In pediatric populations, exposures such as indoor and outdoor pollutants, aeroallergens, tobacco smoke, and inadequate ventilation are strongly linked to poor asthma control and frequent exacerbations [1]. Reducing these exposures is essential to improve disease outcomes, minimize pharmacologic dependence, and promote long-term respiratory health [1,2].

## 2. Materials and Methods

We conducted a non-systematic review of the most relevant studies on “contemporary environmental risk factors in childhood asthma” using the PubMed and Cochrane Library databases, covering publications from January 1990 to November 2024. The articles were analyzed using the following Medical Subject Headings (MeSH) terms and relevant text words, including their synonyms, combinations and truncated variants: “environmental risk factors”, “tobacco smoke”, “air pollution”, “outdoor air pollutants”, “indoor air pollutants”,” indoor allergen”, “outdoor allergen”, “viral infection”, “gut microbiome “, “sedentary lifestyle”, “obesity”, “diet”, “pediatric asthma”. The initial search yielded 31,807 records. After removing duplicate articles, the abstracts of the remaining studies were screened for relevance to the scope of this narrative review. The full texts of relevant papers were then reviewed. Studies that reported outcomes from case reports, case series, case–control studies, synthesized data, cohort studies, randomized controlled trials, and reviews were considered eligible for inclusion. Only articles published in English were included. Exclusion criteria encompassed studies available only as abstracts, letters, editorials, discussion papers, conference proceedings, and animal studies. The selection process began with title screening, followed by abstract review, and concluded with full-text analysis. Titles and abstracts were independently assessed by two reviewers (A.G.L. and M.D.M.). Three independent reviewers (E.D.M., E.A., and F.T.) carried out quality assessments under the supervision of two additional authors (F.I. and L.P.). All data were independently validated. Following the initial search and based on predefined key questions and eligibility criteria, 109 studies were identified as meeting the inclusion criteria.

## 3. Demonstrated Environmental Risk Factors Related to Pediatric Asthma

### 3.1. Tobacco and E-Cigarette Smoke

#### 3.1.1. Tobacco Smoke

Tobacco smoke plays a significant role in the onset of asthma and is a well-known modifiable risk factor for asthma control and prevention of exacerbations. Smokers exhibit significantly higher concentrations of matrix metalloproteinase (MMP)-12 in sputum compared with non-smokers [16]. These levels are inversely correlated with lung function and positively associated with sputum neutrophil counts [16]. MMP-12 is a neutral endopeptidase primarily degrading extracellular matrix components, a physiological process essential for tissue remodeling, growth, and repair [16]. Excessive MMP activity and the imbalance between MMPs and their endogenous regulators, the tissue inhibitors of metalloproteinases (TIMPs), have been implicated in tissue-destructive processes associated with chronic pulmonary diseases, including COPD and asthma [16]. In this context, additional findings have shown reduced MMP-9 activity and lower MMP-9/TIMP ratios in asthmatic smokers compared with non-smokers [16]. Such alterations were associated with persistent airflow obstruction and reduced airway lumen area on CT imaging, suggesting that disruption of the MMP-9/TIMP balance may contribute to structural airway changes in this population [16]. Collectively, these results indicate that chronic exposure to cigarette smoke promotes additive or synergistic inflammatory and remodeling responses in the asthmatic airway [17]. Furthermore, bronchial biopsy specimens from asthmatic smokers demonstrate significantly reduced numbers of CD83+ mature dendritic cells and B lymphocytes compared with asthmatic non-smokers [18]. This may contribute to this group’s higher frequency of lower respiratory tract infections [18]. In general, tobacco smoke can be categorized into two types: first-hand smoke, referring to the aerosol directly inhaled by the smoker, and second-hand smoke (SHS), referring to the aerosol released into the surrounding air from burning tobacco products not directly inhaled [19]. It is known that children passively exposed to cigarette smoke have shown a higher frequency of asthma and wheezing [20]. A meta-analysis demonstrated that the presence of even one smoking parent increased the risk of asthma and wheeze in children by approximately 40% during childhood [21]. More recently, the systematic review based on eight cohort studies by Vork et al. on domestic smoke exposure found a 33% increased risk of asthma incidence during childhood [22]. In recent years, several studies have provided more detailed estimates regarding the effects of prenatal maternal smoking exposure and postnatal maternal, paternal, or domestic smoking on the risk of asthma and wheezing at different pediatric ages. Both prenatal and postnatal maternal smoking exposure are significantly associated with an increased risk of developing asthma and wheeze in children across various age groups [22]. In particular, the most significant findings concerned the association between maternal smoking during pregnancy and the risk of developing asthma in children under two years of age (OR = 1.85, CI 95%= 1.35–2.53) [20]. This risk progressively decreased with the child’s age, although it remained significantly elevated between 5 and 18 years (OR = 1.23, CI 95%= 1.12–1.36) [20]. Postnatal maternal smoking exposure was also associated with a significantly increased risk of newly diagnosed asthma between the ages of 5 and 18 years (OR = 1.20, CI 95% = 0.98–1.46) [20]. Data on postnatal paternal smoking exposure were more limited: only one study conducted on children aged 3–4 years showed a significant result (OR = 1.34, CI 95% = 1.23–1.46) [20]. The exposure to domestic passive smoke was not significantly associated with asthma incidence in children aged ≤2 years (OR = 1.14, CI 95% = 0.94–1.38), but was significantly associated in children aged 3–4 years (OR = 1.21, CI 95% = 1.00–1.47) and 5–18 years (OR = 1.30, CI 95% = 1.04–1.62) [20]. Tobacco smoke exposure, both active and passive, represents a major modifiable risk factor for the development and exacerbation of asthma across all age groups, through mechanisms that promote inflammation, impair immune responses, and disrupt tissue remodeling.

#### 3.1.2. E-Cigarette Smoke and Vaping Exposure

Although electronic cigarettes (e-cigarettes) are frequently viewed as a less harmful alternative to combustible tobacco smoking, emerging evidence suggests a significant relationship between their use and a higher prevalence of chronic respiratory symptoms and asthma incidence in the pediatric population. Recent studies report that both active and passive exposure to e-cigarette aerosol can induce airway inflammation, pulmonary dysfunction, and bronchoconstriction [23,24,25,26,27]. Cho et al.’s study evaluated 35,904 adolescents in South Korea and demonstrated a significant increase in medically diagnosed asthma among e-cigarette users compared to non-users within the 12 months preceding the study (OR 2.36; CI 95% = 1.89–2.94) [23]. Schweitzer et al. conducted a longitudinal study involving 6089 high school students in Hawaii, demonstrating that current e-cigarette use was associated with a current asthma diagnosis, independent of confounding variables such as conventional cigarette smoking, marijuana use, and various socioeconomic factors (OR 1.48; CI 95% = 1.26–1.74) [24]. Although the role of vaping in the pathogenesis of asthma has not been directly examined, substantial evidence suggests that several toxic agents present in e-cigarette aerosols—including formaldehyde, acrolein, acetaldehyde and benzaldehyde—may contribute to asthma-related pathogenic processes [25,26,27]. Thus, despite their perception as a safer alternative, current data indicate that e-cigarette use poses a significant and independent risk for asthma in youth, warranting caution and further investigation into their long-term respiratory effects.

### 3.2. Air Pollution

#### 3.2.1. Outdoor Pollution

Air pollution is defined as the presence in the air of unsafe substances to human health by the World Health Organization (WHO) [28]. Moreover, these substances are associated with a high risk for several diseases (e.g., cardiovascular diseases, cancer, chronic obstructive pulmonary disease, asthma and lower respiratory infection) and they increase the risk of premature death [28]. Polluted air composition differs between seasons and meteorological events, and human activities could influence it [29]. According to WHO’s data, 9 out of 10 people breathe polluted air and more than 80% are exposed to excessive air pollutants [28]. There is always more evidence that indicates that air pollution (outdoor and indoor pollution) contributes to asthma development. GINA’s evidence reports that 13% of the global incidence of asthma in children could be ascribed to traffic-related air pollution (TRAP) [1,2]. Moreover, among TRAP, the components Particulate Matter (PM) 2.5 (OR 1.03, CI 95%), PM_10_ (OR 1.0, CI 95%), NO_2_ (OR 1.05, CI 95%) and black carbon (OR 1.08, CI 95%) have an important role in asthma development [30]. Exposure to polluted air changes the integrity of the epithelium, particularly the expression of pro-inflammatory cytokines, because of activation of Toll-like and Nucleotide-binding Oligomerization Domain (NOD-receptors), and epithelial growth factor receptor [31]. Moreover, exposure to pollutants causes the production of reactive oxygen species (ROS) that attract neutrophils [32]. Production of ROS is also stimulated in the case of Ozone (O3) exposure, which causes a change in claudins’ expression, resulting in permeability of the tight junction [33]. Another evidence underlines that repeated exposure to O3 stimulates group 2 innate lymphoid cells (ILC2)-mediated airway and the nonatopic asthma phenotype [34]. Two recent studies from New York City have highlighted epigenetic changes in immune genes after black carbon exposure [35,36]. Elevated exposure to black carbon results in reduced DNA methylation levels in the IL-4 gene, which may contribute to increased gene expression [36]. A birth cohort study evaluates fine particulate exposure during pregnancy and infancy and the incidence of asthma [37]. Time windows were gestational weeks 6 to 22 (coincided with stages of lung development) and 9 to 46 weeks after birth [37]. Results showed that maternal exposure to PM 2.5 greater than 93 mg/m^3^ might increase the risk of the development of asthma in their children [37]. Moreover, postnatal exposure to PM 2.5 was associated with increased HR of asthma [38]. More than 85% of alveoli are formed after birth, and alveolar formation is completed around 6 months; for this reason, children are susceptible to the adverse effects of air pollution [38]. In summary, the evidence compellingly links both prenatal and postnatal exposure to various air pollutants to the development of asthma through many interconnected pathways, including oxidative stress, epithelial barrier disruption, pro-inflammatory signaling, and epigenetic alterations of immune genes.

#### 3.2.2. Indoor Pollution

Indoor pollution has an important role in the development of asthma, and several factors influence the composition of indoor air [39]. Specifically, factors such as the adequacy and volume of ventilation, the presence of indoor allergens and activities including smoking, heating and cooking, play a crucial role [39]. Increased levels of PM10 and PM 2.5 in the indoor environment have been linked to a higher frequency of severe asthma exacerbations, respiratory symptoms, increased use of asthma medications and more frequent emergency department visits among groups of asthmatic patients (OR = 1.12; 95% CI, 1.04 to 1.22) [40,41]. As one of the main contributing factors to indoor air pollution, biomass combustion is among the most significant [42]. Another important factor associated with high indoor air pollution levels is cooking, particularly using biomass materials (wood, animal dung and crop residues) or coal to cook [32]. Indoor NO_2_, nitrous acid and CO are primarily produced by unflued gas heaters (UFGHs), and they can also make other harmful substances such as formaldehyde [43,44]. Moreover, respiratory symptoms (wheezing and dyspnea) increase in people with UFGHs exposure compared to people without it [45,46]. Air filters may reduce exposure to fine particles; however, their impact on asthma outcomes remains inconsistent [45,46]. Therefore, indoor air pollution represents a significant and modifiable environmental risk factor for asthma exacerbations and morbidity, with sources ranging from household activities like cooking and heating to inadequate ventilation, highlighting the need for targeted public health interventions.

### 3.3. Allergens

#### 3.3.1. Indoor Allergens

Exposure to indoor allergens represents a major modifiable risk factor for the development and exacerbation of pediatric asthma [1,47]. Many studies have shown that both sensitization to allergens and prolonged exposure to allergens such as house dust mites (HDM), mold, cockroaches, and rodents significantly increase asthma risk in children [47]. House dust mite allergens, when present at levels >10 µg/g in early life, are associated with an increased likelihood of asthma onset by age six (OR: 1.8; 95% CI: 1.3–2.6) [48]. Similarly, visible mold exposure in the home environment has been linked to a 56% higher risk of developing asthma (OR: 1.56; 95% CI: 1.19–2.05) [49]. High indoor humidity further elevates the risk (OR: 1.3–1.5), possibly by promoting microbial growth and allergen persistence [50]. School-based exposure is equally critical [51,52]. The SICAS study reported a strong dose–response relationship between mouse allergen (Mus m 1) levels in classrooms and asthma morbidity, with integrated pest management reducing symptoms by 63% [51,52]. Cockroach allergens (Bla g 1, Bla g 2) were associated with increased Th2 responses and worsened asthma control [51,52]. In conclusion, both home and school indoor allergen exposures—particularly from HDM, mold, mice, and cockroaches—are independently and strongly associated with increased pediatric asthma risk and morbidity [53]. Allergen-specific immunotherapy (AIT) may represent a valuable treatment option in clinical contexts where allergy plays a prominent role, including asthma associated with allergic rhinoconjunctivitis [54,55,56,57]. In patients with asthma and allergic sensitization, AIT has been associated with significant reductions in symptom scores and medication requirements, while also improving both allergen-specific and nonspecific bronchial hyperresponsiveness [54,55,56,57]. Thus, pediatric asthma management necessitates a dual strategy: implementing environmental controls to reduce exposure to key indoor allergens and considering AIT as a targeted therapeutic intervention to modify the underlying allergic response in sensitized individuals.

#### 3.3.2. Outdoor Allergens

Pollen is a major outdoor aeroallergen implicated in the exacerbation and potential development of pediatric asthma [1,58]. A 2020 systematic review and meta-analysis by Shrestha et al. included 12 studies, focusing on ambient pollen exposure and asthma hospital admissions in subjects under 18 [58]. The meta-analysis of case-crossover studies found a statistically significant association between grass pollen and asthma hospitalization: 10 grass pollen grains/m^3^ were associated with a 3% rise in asthma admissions (OR = 1.03, CI 95% = 1.01–1.04) [58]. Similarly, for birch pollen, the pooled analysis of time-series studies showed a mean percentage change (MPC) in daily asthma admissions of 0.85% (CI 95% = 0.40–1.30) for every 10-grain/m^3^ increase [58]. Geographic and taxonomic variability was observed across studies [59,60]. While grass and birch pollen showed consistent associations, data for tree pollen (other than birch) were inconclusive [59,60]. Some studies identified significant associations only in specific seasons (e.g., spring), age groups (e.g., children aged 2–12), or during co-occurring meteorological events like thunderstorms [59,60]. For example, in a Sydney study, children aged 2–12 were particularly vulnerable to high levels of conifer, total tree, and total pollen, especially during peak seasons [59]. Another study in Adelaide reported increased asthma admissions during the cool season (April–September) associated with total pollen concentrations, although species-specific data were limited [60]. In conclusion, ambient grass and birch pollen are consistent environmental triggers for asthma hospitalizations in children, with clear seasonal and age-specific patterns [59,60]. These findings support the integration of pollen surveillance and early warning systems into asthma management protocols for pediatric populations [61]. Mold sensitization is key in asthma exacerbations, increased emergency room visits, and respiratory distress [61]. *Alternaria alternata* is one of the most clinically significant fungal aeroallergens worldwide and is strongly associated with the development, persistence, and severity of asthma [62,63]. Exposure occurs predominantly in outdoor environments during late summer and early autumn but can also happen indoors [62]. In the research by Soffer et al., sensitization to *A. alternata* was linked to a 3.7-fold increased risk of frequent wheezing and other asthma symptoms [63]. In the research by Gergen et al., the risk of self-reported asthma in sensitized individuals showed an adjusted odds ratio of 2.3 (CI 95%: 1.5–3.4) [64]. A particularly alarming finding comes from the study by O’Hollaren et al.: it reported an odds ratio of 189 (95% CI: 6.5–5535.8) for episodes of asthma-related respiratory arrest in sensitized patients during the peak season [65]. Treatment with grass pollen AIT for three years did not decrease the incidence of asthma diagnosis in a large randomized, double-blind, placebo-controlled trial involving children aged 5–12 years with grass pollen-induced allergic rhinoconjunctivitis [66]. However, the intervention was associated with a reduction in both asthma symptoms and the use of asthma medications [66]. Therefore, pollen and mold spores represent significant outdoor environmental triggers for severe pediatric asthma exacerbations, necessitating vigilant monitoring and management. However, the role of allergen immunotherapy in preventing asthma onset remains unclear. At the same time, their role in improving asthma control is well demonstrated.

### 3.4. Viral Infections

Early-life viral respiratory infections, particularly by rhinovirus (RV) and respiratory syncytial virus (RSV), have been extensively investigated for their role in the pathogenesis of asthma [67,68,69]. Both viruses are highly prevalent in infancy and associated with recurrent wheeze and the later onset of asthma [67,68,69]. RV infections, especially with RV-A and RV-C species, are potent inducers of wheezing in early childhood [67,68,69]. Epidemiological studies indicate that children with RV-induced wheezing are at significantly higher risk for asthma development [67,68,69]. In the COAST study, RV wheezing episodes during the first 3 years of life were associated with an increased risk of developing asthma at age 6 years (OR 9.8, CI 95% = 3.7–26.0) [69]. This risk was even higher when RV wheezing co-occurred with atopy (OR 26.6, CI 95% = 8.3–85.7) [69]. RSV, the primary cause of bronchiolitis and lower respiratory tract infection in infants, is also strongly associated with subsequent asthma [68]. A systematic review conducted by Feldman et al. indicated a dose–response relationship, with severe RSV infections conferring a greater asthma risk [68]. In one randomized controlled trial, prophylaxis with palivizumab reduced wheezing episodes in preterm infants by nearly 50% during the first year (11% vs. 21%, p = 0.01) [67]. However, its long-term effect on physician-diagnosed asthma remains inconclusive [67]. Both viruses are implicated in the disease’s pathophysiology by innate immunity responses [70,71]. RSV shifts the immune system toward a Th2 profile, reducing IFN-γ (Interferon gamma) production and enhancing airway hyperresponsiveness [70]. Similarly, RV triggers a Th2/ILC2 (Group 2 Innate Lymphoid Cells) -dominant response, leading to eosinophilic inflammation and mucus overproduction, especially in genetically predisposed or atopic children [70]. In conclusion, early-life infections with RV and RSV are significant, independent risk factors for pediatric asthma, with RV-induced wheezing being the more predictive marker when combined with allergic sensitization [70]. The influenza virus contributes to a proportion of asthma exacerbations and represents a significant cause of morbidity and mortality in the general population [71]. A recent systematic review and meta-analysis reported that annual influenza vaccination reduces the risk of asthma exacerbations [71]. However, the possibility of residual bias within the included studies cannot be excluded [71]. Therefore, viral respiratory infections play a dual role in pediatric asthma, with RV and RSV being key drivers of its initial development through specific immune mechanisms, while influenza acts as a major trigger for exacerbations, a risk that can be mitigated through vaccination.

### 3.5. Microbiome

Several studies show a relationship between diversity in the gut microbiome and asthma development, although the precise communication mechanism in the gut-lung axis is poorly understood [72,73,74,75]. An important factor influencing asthma development is lower diversity in the gut microbiota and an excess of Moraxella in the upper respiratory tract (OR 4.52, CI 95%) [72,73]; by contrast, an increase in microbiota diversity is a protective factor [72,73]. The factors influencing gut microbiota include smoke exposure, antibiotics or diet that led to an altered systemic and local immune response, and inflammatory lung changes [74,75]. A review showed that early postnatal life is a crucial moment in the newborn’s life [76]; if this transition is damaged, the risk of atopic disease, including asthma, is higher [76]. Lehtimäki et al. demonstrated a difference between the gut microbiota of urban and rural infants [77]. According to the authors, the microbiome of children from urban areas increases the risk of asthma development, probably through interactions with the nascent immune system [77]. In a randomized controlled trial, Nieto et al. demonstrated that sublingual administration of an inactivated polybacterial mucosal vaccine (MV130—a mixture of six inactivated bacteria)—significantly reduced the incidence of wheezing attacks compared to the placebo group [78]. Recent advances demonstrate that the indoor microbiome and its metabolites critically influence asthma development in children [79,80,81]. High-throughput analyses have revealed that protective metabolites, including flavonoids, indoles, and keto acids, are enriched in environments associated with lower asthma prevalence [79,80,81]. At the same time, synthetic chemicals and mycotoxins are disproportionately detected in homes of asthmatic children [79,80,81]. These metabolites exert immunomodulatory effects, such as suppressing NF-κB (Nuclear Factor kappa-light-chain-enhancer of activated B cells)– mediated inflammation [80]. In contrast, harmful compounds like pelargonic acid and trichothecene mycotoxins induce pro-inflammatory cascades [81]. Machine learning models consistently showed that indoor metabolites and chemicals outperform microbial taxa in predicting asthma and allergic rhinitis, achieving classification accuracies exceeding 74%, compared to ~50% for microbial indicators [80]. Such evidence indicates that metabolites and chemicals are not merely by-products of microbial activity but also direct environmental predictors of pediatric asthma [80]. Their reproducibility across cohorts in Malaysia and China suggests a potential for developing universal metabolite-based biomarkers that transcend geographical variability [79]. Moreover, the consistent enrichment of indole derivatives and flavonoids in low-wheeze environments highlights a plausible protective mechanism involving microbial metabolite interactions that support immune tolerance [80]. Conversely, detecting pesticides, phthalates, and industrial chemicals in high-wheeze schools emphasizes the pathogenic potential of synthetic compounds in driving airway inflammation [80]. In summary, the developing immune system is profoundly shaped by microbial and metabolic exposures, with low diversity in the gut and a deficit of protective metabolites in the indoor environment acting as key determinants of asthma risk, highlighting a move beyond microbes alone to a broader ‘exposome’ approach in understanding disease pathogenesis.

### 3.6. Obesity and Sedentary Lifestyle

Childhood obesity is a significant risk factor for chronic diseases [82]. The link with the development of asthma is bidirectional; if, on one hand, obesity is a predisposing risk for asthma, on the other, patients affected by asthma have a higher risk of developing obesity [82,83,84]. The mechanism behind the impact of obesity on asthma is not clear, but epidemiological data focus on preschool years as a susceptible window for long-term asthma outcomes [85]. A delicate age group is under 6 years of age, as it has been observed that adiposity in this period is predicted to lead to childhood asthma [85]. Similarly, in the Avon Longitudinal Study of Parents and Children, the influence of high BMI in early life on asthma development by the age of 7 years was demonstrated [86]. Childhood obesity is a significant risk factor for asthma, doubling its incidence compared to normal-weight peers [86]. The authors proposed as potential underlying pathophysiological mechanisms both mechanical factors (reduced lung volumes, dysanapsis), metabolic dysregulation (insulin resistance, dyslipidemia), and chronic low-grade inflammation from excess adipose tissue [86]. Furthermore, obesity alters immune responses—shifting toward Th1/Th17 and increasing pro-inflammatory cytokines and adipokines (e.g., leptin, IL-6)—which worsen asthma severity and reduce responsiveness to standard anti-inflammatory therapies [87]. Childhood obesity is closely linked to the concept of a sedentary lifestyle, even if further longitudinal studies are needed to establish its possible role in the development or worsening of pediatric asthma, as current evidence is still unclear [87]. Eijkemans et al., in their study based on a large prospective cohort involving 1838 patients, found no evidence that low physical activity or high sedentary time in early childhood directly increases the risk of developing asthma by school age [88]. Objective accelerometer data showed that children who later developed asthma had similar activity levels compared to children without asthma [88]. Instead, respiratory symptoms or reduced lung function before diagnosis may limit activity, suggesting reverse causation [88]. Based on their results, sedentariness appears more likely to be a consequence, rather than a cause, of pediatric asthma onset [88]. Nevertheless, it is well established that reducing obesity and sedentary behavior in children can significantly decrease the risk of developing chronic diseases and promote healthy growth [89,90]. Therefore, although physical activity may trigger asthma exacerbations in children, especially activities causing prolonged, rapid breathing (e.g., running, soccer, hockey), and respiratory symptoms occurring during exercise could represent a manifestation of underlying latent asthma, sports participation should absolutely not be contraindicated in these patients [91]. Indeed, weight loss improves asthma control, lung function, and quality of life, making body weight management a key therapeutic target and physical activities should be encouraged, as they improve fitness, social participation, and quality of life without worsening lung function [87,91]. In conclusion, childhood obesity is a major modifiable risk factor for asthma, driven by mechanical, metabolic, and inflammatory pathways. While sedentariness may be a consequence rather than a cause, weight management through encouraged physical activity remains a cornerstone of improving asthma control and overall health in affected children.

### 3.7. Diet

A healthy diet represents a protective factor for chronic diseases, such as asthma [83]. Breastfeeding is the first important step because it is associated with a lower risk of developing obesity and asthma [92,93]. Western diet patterns are often rich in saturated fatty acids, low in fiber and high in sugars; this pattern promotes obesity and diseases like asthma [92,93]. In a randomized controlled trial, Wood et al. demonstrated that a single meal rich in saturated fatty acids increases neutrophilic airway inflammation and decreases bronchodilator responsiveness [94]. On the other hand, the Mediterranean Diet (MD) is characterized by a high intake of fruits, vegetables, cereals and olive oil and exhibits antioxidant and anti-inflammatory properties attributable to its richness in micro- and macronutrients, including vitamins (A, C, D), minerals (iron, zinc, selenium, folic acid), and fatty acids (monounsaturated and omega-3 polyunsaturated) [95,96]. In a systematic review and meta-analysis, Hosseini et al. found that fruit and vegetable consumption exerted a protective effect against both the development and exacerbation of asthma [97]. Specifically, vegetable intake was associated with a lower risk of asthma onset, while fruit consumption was inversely correlated with disease severity [97]. Moreover, dietary supplementation with omega-3 polyunsaturated fatty acids (n3PUFAs) may inhibit the production of leukotrienes and other pro-inflammatory mediators [98,99]. However, there is no strong evidence supporting n3PUFA supplementation as a protective factor for developing or controlling pediatric asthma. In a 24-week randomized controlled trial assessing the effects of n3PUFA supplementation on symptoms in overweight adolescents and young adults with uncontrolled asthma, Lang et al. found no significant improvements in asthma control or pulmonary function at either 3–6 month [100].

### 3.8. Synergistic Effects of Multiple Environmental Exposures

Potential contemporary environmental risk factors for childhood asthma are well-demonstrated when analyzed individually (Table 1).

At the same time, potential contemporary environmental risk factors for childhood asthma often exhibit synergistic effects [79]. Several studies suggest that air pollution represents a significant environmental determinant in developing allergic diseases such as asthma, rhinitis, and eczema [79]. Between 2012 and 2019, a significant increase in the prevalence of eczema symptoms was observed (from 3.6% to 7.0%; p < 0.001), while asthma showed a borderline significant increase (p = 0.06) and rhinitis remained stable [79]. In parallel, exposure to air pollutants such as nitrogen dioxide (NO_2_) and particulate matter (PM_10_) was significantly associated with an increase in eczema symptoms (p = 0.02–0.03) [79]. This effect is likely mediated by the impairment of epithelial barrier integrity, which facilitates the penetration of allergens and microorganisms, promoting allergic-type inflammatory responses [79]. Moreover, air pollution appears to modulate the composition of the indoor environmental microbiome [79]. Higher concentrations of NO_2_ and PM_10_ were associated with the presence of bacterial taxa considered protective against rhinitis (e.g., Prevotella, Lactobacillus iners, Dolosigranulum), suggesting a complex interaction between pollutants, microbiota, and the host immune system [79]. However, such alterations may reflect a state of microbial dysbiosis, with unclear long-term health implications [79]. Fungal components of the microbiome were also significantly correlated with allergic conditions [79]. Greater fungal diversity—particularly within Dothideomycetes, Eurotiomycetes, and Sordariomycetes—was positively associated with eczema symptoms (p < 0.05) [79]. Notably, the presence of the mold species Aspergillus subversicolor was significantly associated with asthma symptoms (p = 0.005), indicating that exposure to specific indoor fungal taxa may contribute to the onset or exacerbation of respiratory symptoms [79]. Furthermore, air pollution is known to impair innate immune responses, increasing susceptibility to viral respiratory infections [79]. Fine particulate matter (PM_10_ and PM_2_._5_) can disrupt the function of epithelial cells and dendritic cells, predisposing individuals to chronic inflammatory states that may trigger or worsen allergic diseases, particularly in children undergoing immune system maturation [79].

## 4. Environmental Risk Factors in Pediatric Asthma: Is It Possible to Establish Which Weighs More?

This review deepened various environmental risk factors, including cigarette smoke, air pollution, allergens, respiratory infections, microbiome, obesity, sedentary lifestyle and diet, as potential risk factors for asthma in childhood. Among these factors, our data found that maternal smoking during pregnancy plays a significant role in the onset of asthma in children under two years of age [20,21,22]. Furthermore, house dust mites are a key allergen; exposure to levels exceeding 10 µg/g in early childhood is associated with an increased likelihood of asthma onset by age six [48,49,50]. Another important risk factor contributing to the development of pediatric asthma is viral respiratory infection occurring within the first three years of life, particularly rhinovirus infection, which initially causes wheezing and subsequently leads to asthma by age six [67,68,69,70]. Specifically, Zhou et al. conducted a meta-analysis focused on early-life risk factors and identified strong associations between pediatric asthma and various exposures, including maternal smoking during pregnancy (OR = 1.41; CI 95% = 1.21–1.64), early-life respiratory infections (OR = 2.05; CI 95% = 1.62–2.59), and a family history of asthma [101]. These findings were further confirmed and expanded by Castro-Rodriguez et al., who synthesized 41 high-quality systematic reviews, identifying parental asthma, prenatal exposure to environmental tobacco smoke (ETS), and prematurity—particularly very preterm birth—as consistently strong predictors of childhood asthma development [89]. At the same time, prenatal ETS exposure was correlated with a higher incidence of asthma in different age groups (OR range: 1.28–1.52) [102]. Anyway, no studies have definitively established a hierarchic list of the potential contemporary environmental risk factors for childhood asthma in the context of its onset and control of asthma symptoms.

## 5. The Social and Economic Burden of Pediatric Asthma: Prevention and Mitigation Strategies

Pediatric asthma presents a considerable social impact, affecting the child’s health, education, family relationships and quality of life [103]. Children with asthma often miss school, limiting their academic achievement and social interaction [103]. Meanwhile, parents may experience increased stress and financial strain due to healthcare costs and lost workdays [103]. Additionally, both the child and their family can experience social isolation, stigma, and mental health issues due to the condition [103]. According to Zhang et al., childhood asthma accounts for the greatest disability burden, resulting in nearly 13.8 million school absence days in the United States in 2013 [103]. Furthermore, it has been reported that children with asthma require psychological support, as the condition can negatively impact educational outcomes, potentially leading to lower academic achievement and early school dropout [103]. Globally, childhood asthma is often accompanied by comorbidities such as allergic rhinitis, impaired lung function, and mental health disorders [15]. The burden of childhood asthma is particularly significant in high-income countries [15]. According to Ferrante et al., asthma contributes to approximately 1.1% of the global Disability-Adjusted Life Years (DALYs) per 100,000 population across all causes [15]. In addition, asthma-related costs are considerable and typically categorized into direct, indirect, and intangible costs [15]. Direct costs account for 50–80% of the total costs (the annual costs from U.S. children’s asthma caused by environmental exposures are estimated at $2.3 billion) [15]. Asthma is a leading cause of hospitalization, particularly among children aged <5 years: actually, during the last two decades, its prevalence has increased, mostly in lower-middle-income countries [15]. Indirect costs usually represent a higher burden in older patients, including school and work-related losses [15]. Impairment of quality of life, limitation of physical activities and study performance are some examples of intangible costs, with the result of being unquantifiable [15]. Despite increasing medication costs and an increase in the worldwide prevalence of pediatric asthma, there is evidence that identifying the key modifiable risk factors related to childhood asthma can suggest different prevention strategies, which is key for decreasing childhood asthma development/worsening and reducing its burden [1]. Several studies have already investigated the effects of smoke-free legislations regarding the potential reduction in pediatric asthma onset and severity across different countries: the study from Hatoun et al. found that, in America, a stronger tobacco tax is associated with reduced asthma severity (adjusted odds ratio = 1.40; *p* = 0.007, 95% confidence interval: 1.10–1.80) [104]. These results are consistent with the ones from Lee et al., stating that, after the introduction of smoke-free laws in Hong Kong, a change in the admission count of −33.5% (95% CI −36.4% to −30.5%) was immediately observed, with a net 47.4% reduction in admission counts in the first year [105]. A systematic review and meta-analysis by Radò et al. found that smoke-free car policies are associated with an immediate tobacco smoke exposure (TSE) reduction among children in cars (risk ratio 0.69, 95% CI 0·55–0·87; 161 466 participants); with a possible translation into an estimated 0.2–2.4% decrease in asthma diagnoses [106]. Other potential strategies have been proposed and proved to help reduce the burden of pediatric asthma: simple mechanical home interventions effectively reduce allergen loads in the home, reduce symptoms and urgent care associated with asthma, and prevent disease emergence [107]. Bedding covers, vacuum cleaners, air purifiers, improved ventilation and central heating can reduce indoor air allergens and often lead to better respiratory health in children [107]. Combined interventions incorporating mechanical allergen-reduction methods alongside educational programs for children with asthma and their parents have proven effective in preventing asthma and minimizing exposure to triggers [107]. These strategies have significantly improved health outcomes in children with asthma [107]. Finally, nutritional interventions have also been tested as a possible primary prevention strategy with encouraging results, even if the evidence is insufficient to allow strong recommendations about diet changes to prevent pediatric asthma [108]. Thus, knowing modifiable environmental risk factors for pediatric asthma is essential for effective prevention. Early identification and mitigation of these factors can reduce the incidence of the disease, improve long-term health outcomes in children, and significantly decrease the economic burden associated with asthma care and related healthcare services.

## 6. Conclusions

In recent decades, the prevalence of asthma and allergic diseases has increased significantly, leading to global efforts to identify modifiable risk factors for prevention. Several risk factors for childhood asthma were identified, including tobacco smoke, air pollution, allergens, viral infections, gut microbiome, physical activity, obesity and diet. No studies have clearly and unequivocally determined which among the multiple risk factors weighs more on the onset and control of pediatric asthma. Maternal smoking during pregnancy, passive smoking, viral infection within the first three years of life and indoor allergens, particularly house dust mites, seem to have the most important role (Table 2).

This article emphasizes how the actual risk profile for pediatric asthma is defined not by single factors, but by the interplay between an evolving set of environmental exposures. These contemporary risk factors cover a wide range, from traditional triggers like tobacco smoke and allergens to emerging threats such as e-cigarette vaping, microbiome, and indoor pollution. Establishing a definitive hierarchy remains a challenge. Anyway, a comprehensive and active approach by the pediatric allergist is needed to identify, monitor, and simultaneously address all potential environmental risk factors to ensure optimal asthma control and reduce exacerbation frequency and severity.

## Figures and Tables

**Table 1:** Main studies about risk factors for pediatric asthma.

Author	Year	Risk Factor	Results and Effect Size (Odds Ratio, 95% CI)
Burke et al. [20]	2012	Prenatal maternal smoking	OR = 1.85 (95% CI: 1.35–2.53) for asthma <2 years; OR = 1.23 (95% CI: 1.12–1.36) for 5–18 years
Burke et al. [20]	2012	Postnatal maternal smoking	OR = 1.20 (95% CI: 0.98–1.46) for asthma in 5–18-year-olds
Burke et al. [20]	2012	Postnatal paternal smoking	OR = 1.34 (95% CI: 1.23–1.46) children 3–4 years
Burke et al. [20]	2012	Household second-hand smoke	OR = 1.14 (95% CI: 0.94–1.38) ≤ 2 years (NS); OR = 1.21 (95% CI: 1.00–1.47) 3–4 years; OR = 1.30 (95% CI: 1.04–1.62) 5–18 years
Cho et al. [23]	2016	E-cigarette (active use)	OR = 2.36 (95% CI: 1.89–2.94) (Cho et al.); OR = 1.48 (95% CI: 1.26–1.74) (Schweitzer et al.)
Khreis et al. [30]	2017	Outdoor air pollution (TRAP)	PM2.5: OR = 1.03; PM10: OR = 1.00; NO_2_: OR = 1.05; Black carbon: OR = 1.08 (95% CI)
McConnell et al. [41]	2003	Indoor air pollution	PM2.5/PM10 linked to exacerbations and symptoms; biomass and unflued gas heaters worsen symptoms (OR = 1.12; 95% CI, 1.04 to 1.22)
Celedon et al. [48]	2007	Indoor allergens (HDM, mold, rodents, cockroaches)	HDM >10 µg/g: OR = 1.8 (95% CI: 1.3–2.6); mold: OR = 1.56 (95% CI: 1.19–2.05); high humidity OR = 1.3–1.5; allergen reduction = –63% symptoms
Shrestha et al. [58]	2021	Outdoor allergens (grass and birch pollen, Alternaria)	Grass pollen: OR = 1.03 per 10 grains/m^3^ (95% CI: 1.01–1.04); birch pollen MPC = 0.85% increased admissions; Alternaria OR = 2.3–189
Jackson et al. [69]	2008	Viral infections (RV and RSV)	RV wheezing: OR = 9.8 (95% CI: 3.7–26.0); RV + atopy: OR = 26.6 (95% CI: 8.3–85.7); RSV severe dose–response; palivizumab reduces episodes ~50%
Depner et al. [72]	2017	Gut microbiome	Low diversity and Moraxella overgrowth: OR = 4.52 (95% CI);
Eijkemans et al. [88]	2020	Childhood obesity	Asthma risk doubled compared to normal weight peers; mechanisms: mechanical, metabolic, inflammatory; stronger in <6 years (no specific OR reported)
Hughes et al. [91]	2014	Sedentary lifestyle	No direct evidence that it increases asthma risk; sedentariness is more likely a consequence of asthma
Hosseini et al. [97]	2017	Diet	Western diet (high saturated fat, low fiber) linked to increased risk; Mediterranean diet and fruit/vegetable intake protective; n3PUFA supplements not effective for asthma control

**Table 2:** Main Outcomes and Findings.

Asthma and allergic disease prevalence has risen markedly in recent decades.
Multiple risk factors are implicated: tobacco smoke, air pollution, allergens, viral infections, gut microbiome, obesity, physical inactivity, and diet.
The relative weight of these factors remains uncertain. Strongest evidence implicates: Maternal smoking during pregnancy Passive smoking exposure Viral infections in the first 3 years of life Indoor allergens, particularly house dust mites
Pediatric allergists should adopt a comprehensive, proactive strategy to monitor and mitigate multiple risks simultaneously.
Multifactorial interventions are essential to achieve optimal disease control and reduce exacerbation frequency and severity.

## Data Availability

Not applicable.

## Abbreviations

The following abbreviations are used in this manuscript:

e.g.,	Exempli gratia
TRAP	Traffic Related Air Pollution
CAMP	Childhood Asthma Management Program
GINA	Global Initiative for Asthma
COPD	Chronic Obstructive Pulmonary Disease
SHS	Second Hand Smoke
OR	Odds Ratio
CI	Confidence Interval
WHO	World Health Organization
NOD	Nucleotide binding Oligomerization Domine
ROS	Reactive Oxygen Species
ILC2	Innate Lymphoid Cells Group 2
HR	Hazard Ratio
PM	Particulate Matter
UFGH	Unflued Gas Heater
HDM	House Dust Mite
MPC	Mean Percentage Change
RV	RhinoVirus
RSV	Respiratory Syncytial Virus
MD	Mediterranean Diet
n3PUFAs	omega-3 polyunsaturated fatty acids
ETS	Environmental Tobacco Smoke
DALYs	Disability-adjusted life years
TSE	Tobacco Smoke Exposure
AIT	Allergen-specific Immunotherapy
SCIT	Subcutaneous Immunotherapy
SLIT	Sublingual Immunotherapy
IL-10	Interlekin-10
TGF-β	Transforming Growth Factor-beta
NF-κB	Nuclear Factor kappa-light-chain-enhancer of activated B cells
